# Drug Resistance and Novel Therapeutic Approaches in Invasive Candidiasis

**DOI:** 10.3389/fcimb.2021.759408

**Published:** 2021-12-14

**Authors:** Sarah E. Murphy, Tihana Bicanic

**Affiliations:** ^1^ Institute of Infection & Immunity, St George’s University of London, London, United Kingdom; ^2^ Clinical Academic Group in Infection and Immunity, St. George’s University Hospital National Health Service (NHS) Foundation Trust, London, United Kingdom

**Keywords:** *Candida*, invasive candidiasis, candidaemia, antifungal resistance, antifungal drugs, drug therapy, drug resistance, antimicrobial resistance

## Abstract

*Candida* species are the leading cause of invasive fungal infections worldwide and are associated with acute mortality rates of ~50%. Mortality rates are further augmented in the context of host immunosuppression and infection with drug-resistant *Candida* species. In this review, we outline antifungal drugs already in clinical use for invasive candidiasis and candidaemia, their targets and mechanisms of resistance in clinically relevant *Candida* species, encompassing not only classical resistance, but also heteroresistance and tolerance. We describe novel antifungal agents and targets in pre-clinical and clinical development, including their spectrum of activity, antifungal target, clinical trial data and potential in treatment of drug-resistant *Candida*. Lastly, we discuss the use of combination therapy between conventional and repurposed agents as a potential strategy to combat the threat of emerging resistance in *Candida*.

## 1 Introduction

*Candida* species are commensal yeasts of the skin, gastrointestinal tract, and other mucosal surfaces of healthy humans. These opportunistic pathogens do not pose a risk to healthy individuals yet have the potential to cause invasive infections in the context of local or generalised reduction in host immune defences or antibiotic-induced overgrowth. Invasive candidiasis (IC) refers to a group of diseases initiated by *Candida* species that comprises candidaemia (bloodstream infection), disseminated and deep-seated (abdominal) candidiasis (Pappas et al., 2018). Increasing use of broad-spectrum antibiotics, an ever-expanding range of immunosuppressive disease states (e.g. HIV/AIDS) and treatments (e.g. for cancer and following solid organ transplantation), and advances in intensive care medicine have led to rising incidence of IC over the past two decades. IC now represents the fourth most common cause of nosocomial bloodstream infections and the most common invasive fungal infection in the UK (Pegorie et al., 2017). IC is associated with a high mortality rate (40-60%) leading to an estimated 400,000 deaths globally each year (Zaoutis et al., 2005; Pappas et al., 2018).

*Candida albicans, Candida glabrata, Candida parapsilosis and Candida tropicalis* are the four *Candida* species most frequently isolated from IC cases (Toda et al., 2019). Although variation exists based on age and geography, most likely due to differences in antifungal usage and species background, *C. albicans* remains the most frequently clinically isolated species, however the past decade has witnessed an increase in the proportion of IC caused by non-*albicans* species (Pfaller et al., 2011b; Castanheira et al., 2016).

**Figure 1:**
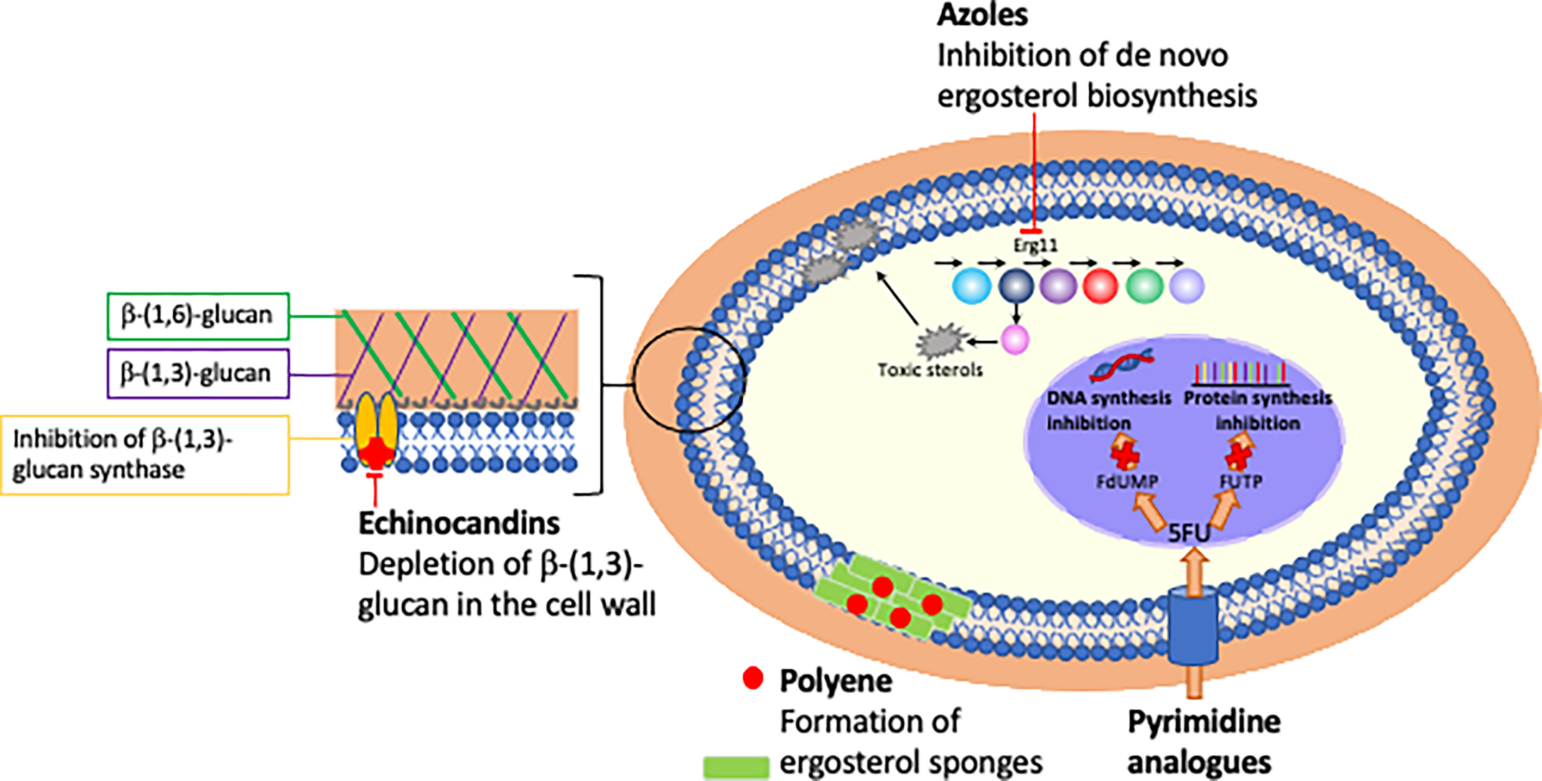
Mechanism and site of action of currently licensed antifungal drugs.

Just three classes of antifungals targeting two unique pathways only are used as first-line treatment of IC (Pappas et al., 2015). The polyenes (e.g. amphotericin B) and the azoles (e.g. fluconazole) both target the major fungal sterol ergosterol, whilst the echinocandins (e.g. anidulafungin) disrupt the fungal cell wall through inhibition of β-1,3-glucan synthase (**Table 1**; **Figure 1**). In contrast to bacteria where 15 new antibiotics representing 5 novel drug classes were approved for use in the past two decades (Hutchings et al., 2019), just one new antifungal class has been clinically deployed during this timeframe: the echinocandins have been the only new antifungal drug class approved for use in IC since the early 2000s (Letscher-Bru and Herbrecht, 2003) and the azole isavuconazole is the only antifungal approved for IC in the past decade (Miceli and Kauffman, 2015). Due to their fungicidal activity and favourable safety profile in clinical trials, the echinocandins are now the first-line antifungal treatment for IC in clinical guidelines (Pappas et al., 2015).

**Table 1:** Antifungal drug classes, targets and frequently observed mechanisms of resistance.

Drug class	Target pathway	Drug target	Mechanism of action	Mechanism of resistance	Species with reported resistance
Azoles (fluconazole, voriconazole, itraconazole, posaconazole, isavuconazole)	Cell membrane (Ergosterol)	Erg11p (lanosterol 14-α-demethylase)	Inhibits *de novo* ergosterol synthesis thereby depleting membranes of ergosterol and causing accumulation of toxic sterol precursors	Increased drug efflux Mutations in Erg11p Overexpression of Erg11p Copy number variation Incorporation of non-ergosterol sterols into cell membranes	*C. albicans* *C. glabrata* *C. tropicalis* *C. dubliniensis* *C. parapsilosis* *C. krusei* (intrinsic) *C. auris* (almost universal)
Echinocandins (caspofungin, anidulafungin, micafungin)	Cell wall (β-1,3-glucan)	β-1,3-glucan synthase	Inhibits β-1,3-glucan synthesis thereby disrupting cell wall stability	Mutations in FKS1/2	*C. albicans* *C. glabrata* *C. auris*
Polyenes (amphotericin B)	Cell membrane (Ergosterol)	Sterols (ergosterol)	Major: Sequesters ergosterol out of membranes. Minor: induces pore formation causing ion leakage	Incorporation of non-ergosterol sterols into cell membranes	*C. albicans* *C. glabrata* *C. guillermondii* *C. krusei* *C. lusitaniae* *C. auris*
Pyrimidine analogues (5- fluorocytosine)	DNA synthesis, Protein synthesis	FUMP, FDUMP	Inhibits pyrimidine metabolism	Mutations in UPRT, FCY1, FCY2, FUR1	*C. albicans* *C. glabrata*

The emergence of antifungal resistance remains an ever-present threat to the limited antifungal armamentarium. The paucity of antifungal drug classes, coupled with the intrinsic plasticity of the fungal genome promotes fungal adaptation and survival under antifungal drug stress. An increasing number of *Candida* species that are resistant to first line antifungal treatments (azoles or echinocandins) are being identified (Pfaller et al., 2011b; Castanheira et al., 2016), particularly in high antifungal use settings thereby almost eliminating all current treatment options (Healey et al., 2016). This trend is paralleled by increased clinical prevalence of multi-drug resistant isolates (e.g. azole and echinocandin resistant *C. glabrata*).

*Candida auris* has emerged as a global pathogen over the past decade, reported from all continents except Antarctica, with the majority of cases associated with ICU outbreaks and high mortality rates (Chowdhary et al., 2014; Sears and Schwartz, 2017; Vallabhaneni et al., 2017; Lamoth and Kontoyiannis, 2018). *C. auris* is usually intrinsically resistant to fluconazole (93%) with varying resistance to the echinocandins (7%) and polyenes (35%), with 41% of isolates reported as multidrug resistant (Chowdhary et al., 2013; Calvo et al., 2016; Lockhart et al., 2017; Vallabhaneni et al., 2017) and 4% of strains pan-resistant to azoles, polyenes and echinocandins (Lockhart et al., 2017). Unusually for *Candida* species, *C. auris* can spread through person-to-person contact, persisting on surfaces and medical devices for months, potentially due to its ability to form biofilms (Schelenz et al., 2016; Eyre et al., 2018). The high transmissibility of this pathogen is highlighted in reports of hospital outbreaks with clonal isolates (Ruiz-Gaitán et al., 2018).

The increasing clinical prevalence of multidrug resistant *Candida* species such as *C. glabrata* and *C. auris* highlights the potential for fungi to pose a serious future threat if we fail to steward and deploy existing and novel antifungal treatments in a manner that prevents the emergence of resistance. In this review, we discuss mechanisms whereby *Candida* species evade antifungals and identify promising novel drugs and therapeutic strategies to tackle this.

## 2 Mechanisms of Antifungal Resistance

### 2.1 Antifungal Susceptibility Testing

The susceptibility of fungal isolates to antifungal drugs is quantified by determining the minimum inhibitory concentration (MIC) through established CLSI (CLSI, 2017) or EUCAST methods (Arendrup et al., 2012). To guide clinicians with treatment selection, the MIC is compared to predetermined clinical breakpoints specific for a drug-species combination and classifies the isolate as susceptible, intermediate/susceptible, dose-dependent, or resistant. Despite the high mortality rate of IC, resistance in *C. albicans* remains relatively rare to the echinocandins and azoles (~1%) (Pfaller et al., 2019) highlighting the fact that the MIC is one of many factors that govern treatment success or failure in IC. Risk factors such as source control (e.g. removal of prosthetic material/devices associated with biofilm formation), fungal burden, extent and reversibility of host immunosuppression, penetration of antifungals into the site of infection, and the potential for pathogen growth at high MICs that is missed by conventional MIC methods are all important considerations that have been extensively covered in other reviews (Pappas et al., 2018; Berman and Krysan, 2020; Perfect and Ghannoum, 2020).

### 2.2 Routes to Resistance

Resistance has been reported against all antifungal drug classes, however the extent of resistance varies between classes and fungal species. Resistance can be classified as either intrinsic (resistance without prior antifungal exposure, e.g. *C. krusei* and fluconazole) or acquired (developing following antifungal exposure in a previously susceptible isolate, e.g. *C. albicans* and fluconazole) (Dagi et al., 2016). Although the overall rates of resistance remain low, the frequency at which both intrinsically and acquired resistant strains are isolated is increasing.

**Figure 2:**
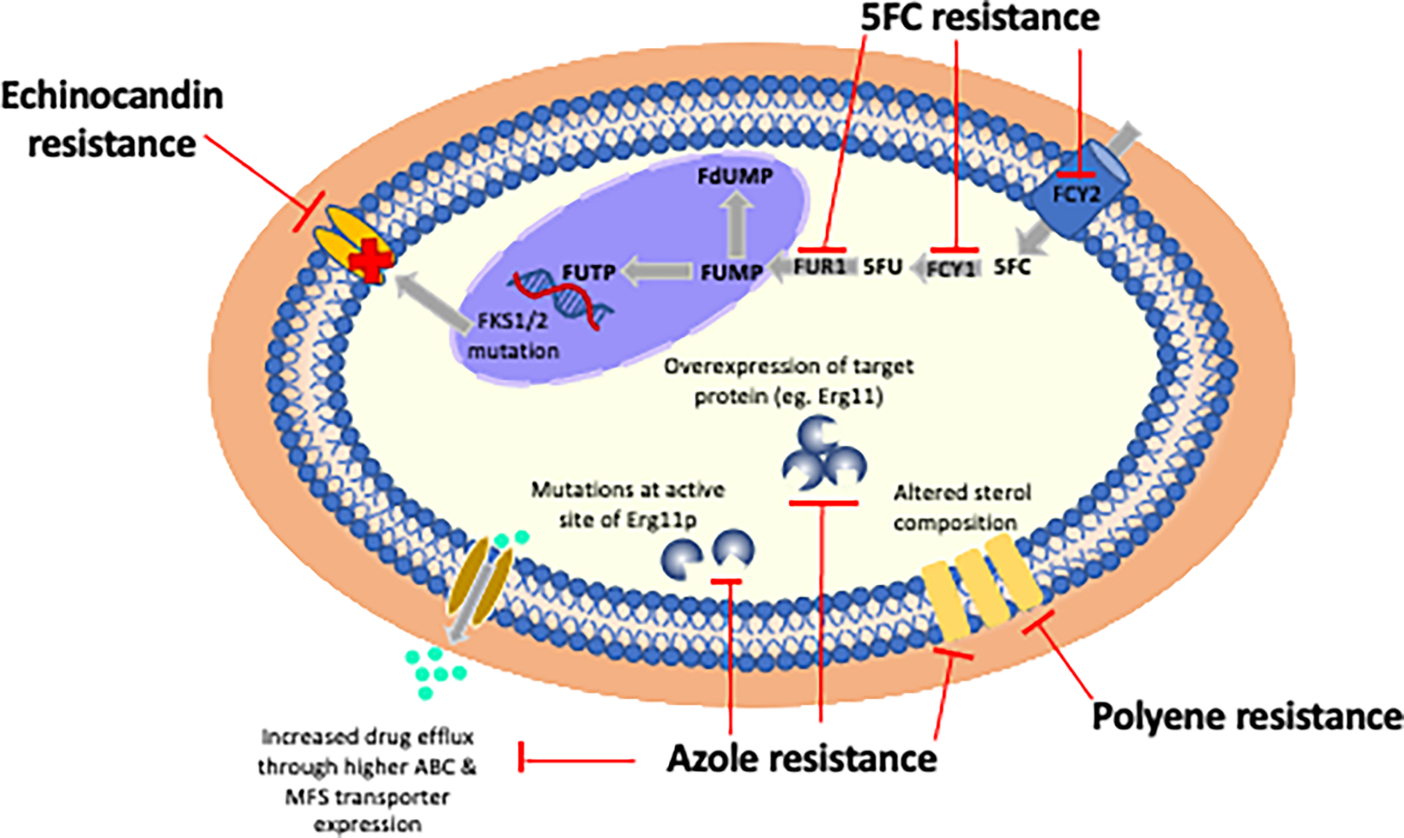
Mechanisms of resistance of currently licensed antifungal drugs. Echinocandin resistance is almost exclusively due to point mutations in three hot spot regions in FKS1 or less frequently due to mutations in FKS2. The most frequently observed mechanism of azole resistance is reduced intracellular accumulation of drug through over-expression of efflux pumps (e.g. ABC or MFS transporters). Polyene resistance is due to incorporation of non-ergosterol sterols into cell membranes. 5FC resistance is mediated by point mutations in enzymes controlling its cellular uptake and conversion to 5FU: cytosine permease (FCY), cytosine deaminase (FCA1), and phosphoribosyl transferase (FUR1).

Resistance can occur through any single or concurrent mechanism (**Figure 2**).

#### 2.2.1 Increased Activity of Drug Efflux Pumps

Increased activity of drug efflux pumps is the most frequently observed mechanism of azole resistance in clinical *Candida* isolates (Sasse et al., 2012; Prasad et al., 2016). Two major efflux families are associated with resistance, the ATP-binding cassette (ABC) transporters and the major facilitator super family (MFS) pumps. Gain-of-function mutations in transcriptional regulators for both families, such as *TAC1* (*C. albicans*) and *PDR1* (*C. glabrata*) increase efflux pump expression and therefore lower intracellular accumulation of drug. These transcriptional regulators have additional non-protein pump targets that further contribute to the development of resistance, such as *GPX1*, a glutathione peroxidase that enhances oxidative stress responses (Rogers and Barker, 2002; Liu et al., 2007) and *CaCHK1*, a histidine kinase which regulates cell wall biosynthesis (Liu et al., 2007; Sasse et al., 2012). A significant portion of the *C. auris* genome is devoted to the ABC and MFS efflux pump families which is consistent with its lack of response to azoles at typical therapeutic doses (Sharma et al., 2016; Ben-Ami et al., 2017; Chowdhary et al., 2018).

#### 2.2.2 Overexpression of Target Protein

Overexpression of target protein due to gain-of-function mutations in transcriptional regulators or gene duplication overwhelms the inhibitory capacity of the drug. Azole resistance in *C. albicans* can be mediated by gain-of-function mutations in key *ERG11* transcriptional regulators, for example A643V in Upc2, resulting in the constitutive overexpression of Erg11 and reduced sensitivity to azoles (Flowers et al., 2012). Overexpression of *ERG11* is also noted in the frequently intrinsically azole resistant species *C. auris*. The *C. auris* genome contains gain-of-function mutations in *TAC1* at comparable regions to azole resistant *C. albicans*, a transient duplication of a region in chromosome 1 that contains *ERG11* (Bhattacharya et al., 2019), and a duplication of the whole of chromosome 5 that contains *TAC1* (Carolus et al., 2021).

#### 2.2.3 Conformational Changes to the Target Protein

Conformational changes to the target protein due to point mutations at or adjacent to the binding site also result in reduced antifungal susceptibility. More than 100 SNPs in *ERG11* that reduce *Candida* species sensitivity to azoles have been described (Flowers et al., 2015). Fluconazole-resistant *C. auris* from multiple continents contain amino acid substitutions in the drug target, Erg11, at known azole-resistant sites (e.g. F126T, Y132F, K143R) that are also present in resistant, but not wild-type *C. albicans* (Lockhart et al., 2017; Chowdhary et al., 2018; Healey et al., 2018). In contrast to azoles where multiple mechanisms of resistance have been identified, resistance to echinocandins in all *Candida* species is almost always due to a very limited number of mutations in *fks1/2* that encodes the echinocandin target enzyme, β-1,3-glucan synthase (Perlin, 2015). Mutation in either of two highly conserved hot spot regions of Fks1 (F641-P649 and R1361) is most frequently the cause of echinocandin resistance and increases the MIC by up to 100-fold (Garcia-Effron et al., 2009). Mutations in equivalent regions of Fks2 have been described in *C. glabrata* (Arendrup et al., 2012; Arendrup and Perlin, 2014; Jensen et al., 2014).

#### 2.2.4 Alterations to Target Pathway

Azoles and polyenes both target the major yeast sterol, ergosterol. Alterations to membrane ergosterol content through accumulation of the precursor sterol 14-α-methyl fecosterol can occur both through inhibition of Erg11 or reduction in the intracellular concentration/activity of this enzyme. Both mechanisms result in functional yeast cells that can bypass the activity of azoles and polyenes *via* altered membrane sterol composition (Haynes et al., 1996; Nolte et al., 1997).

#### 2.2.5 Copy Number Variation

Copy number variation of whole or partial regions of chromosomes, as well as loss of heterozygosity, also confer resistance (Coste et al., 2006; Selmecki et al., 2006; Sasse et al., 2012; Yang et al., 2013; Yang et al., 2019), occurring at least twice as frequently as point mutations (Forche et al., 2011). The left arm of chromosome 5 in *C. albicans* contains both *TAC1* and *ERG11*. Duplication of this portion of the chromosome provides two extra copies of both genes, conferring azole resistance through increasing the amount of drug target whilst simultaneously reducing intracellular drug accumulation (Selmecki et al., 2006). Diploid fungi can undergo loss of heterozygosity such that they become homozygous for alleles containing point mutations associated with resistance. In serial mucosal *Candida* isolates from an HIV-infected patient on fluconazole treatment, loss-of-heterozygosity resulted in homozygosity for a region of chromosome 5 containing a mutant *erg11* allele, and these genetic changes were associated with persistence of infection for 8 months (White, 1997; Ford et al., 2015).

Additional mechanisms of resistance identified in *C. auris* are driven by physiological differences in cell wall content, sterol composition, glycerolipids, and sphinoglipids relative to other *Candida* species (Zamith-Miranda et al., 2019). Moreover, biofilm formation is an important property facilitating the development of resistance through persistence on medical devices such as intravenous catheters, providing a physical barrier to antifungal penetration (Borman et al., 2016; Sherry et al., 2017).

In addition to non-pathogen related factors described above, other fungal adaptive strategies beyond the classical resistance mechanisms may precede and facilitate the emergence of classical resistance. These include subpopulations of cells that are ‘heteroresistant’ or ‘tolerant’ to antifungals. Heteroresistance and tolerance are distinct mechanisms of antifungal drug evasion that are missed by standard clinical MIC testing protocols.

#### 2.2.6 Heteroresistance

Fungi are metabolically and physiologically dynamic which is essential to their adaptation and survival in diverse habitats. There is substantial cell-to-cell variation in gene expression even amongst clonal populations grown at constant environmental conditions. This standing variation, referred to as bet-hedging, is a strategy to increase the likelihood of survival of at least some cells under stress conditions (Levy et al., 2012). Heteroresistance is defined as a subpopulation (<0.1%) within an apparently isogenic, susceptible isolate that has an intrinsically higher MIC compared to the rest of the population. Although heteroresistance has been widely studied in bacteria (Pournaras et al., 2005; Nunes et al., 2006; Morand and Mühlemann, 2007; Hofmann-Thiel et al., 2009), limited attention has been given to its role in fungal resistance with the majority of research undertaken on *Cryptococcus* species (Sionov et al., 2010; Varma and Kwon-Chung, 2010): a PubMed search using the terms ‘heteroresistance’ and ‘yeast’ yielded 24 hits, of which just 2 were related to *Candida* (Claudino et al., 2009; Ben-Ami et al., 2016).

Heteroresistance can be identified using population analysis profiling (PAP) assays whereby isolates are cultured on solid media across a range of drug concentrations (El-Halfawy and Valvano, 2015). Heteroresistance is an adaptive response such that when isolates are serially cultured onto drug, each generation will demonstrate an expansion in the non-susceptible subpopulation of colonies which can then further adapt to grow at higher concentrations of drug (Marr et al., 2001). Heteroresistance is not detected by current susceptibility testing methods. The implications of failing to consider or detect this phenomenon may be clinical failure and persistent or relapsed infection (Ben-Ami et al., 2016).

The variation in intrapopulation drug susceptibility and thus the degree of heteroresistance of each isolate is likely a consequence of both genetic and epigenetic mechanisms. To date, the majority of heteroresistance studies having been performed with azoles and, at least in *Cryptococcus*, primarily occurs *via* the formation of aneuploidies (Sionov et al., 2010; Stone et al., 2019). The fungistatic nature of azoles halts fungal growth and promotes genome instability and consequently the emergence of heteroresistant colonies (Shor and Perlin, 2015). Ben-Ami et al. identified upregulation of the ABC transporter genes *CDR1* and *PDH1* in heteroresistant *C. glabrata* isolates, albeit not to the extent of fully resistant isolates, that was associated with enhanced fluconazole efflux and persistent infection in a murine model (Ben-Ami et al., 2016). Studies in *Cryptococcus neoformans* demonstrated that fluconazole monotherapy in patients with cryptococcal meningitis drove the expansion of aneuploid heteroresistant subpopulations within just two weeks of fluconazole monotherapy. Disomy of chromosome 1, containing *ERG11* and the efflux pump *AFR1*, was most identified as the most common mechanism of heteroresistance (Stone et al., 2019). Importantly, combination therapy of fluconazole with 5FC was sufficient to suppress this resistance emergence. The relevance of heteroresistance in IC, particularly within the context of treatment with fungicidal echinocandins, is unknown. Future work is needed to determine the relevance of heteroresistance in clinical disease progression and the potential of combination therapy to overcome heteroresistance in IC are both fundamental areas to research.

#### 2.2.7 Tolerance

Tolerance is another pathogen factor not detected by the MIC which affects fungal growth *in vitro* and may play a role in treatment response and resistance emergence. Tolerance is distinct from resistance and is defined as a subpopulation of cells within a susceptible isolate that grow and emerge slowly at supra-MIC fungistatic drug concentrations (azoles) (Berman and Krysan, 2020) or survive at supra-MIC fungicidal concentrations (echinocandins) (Healey and Perlin, 2018; Garcia-Rubio et al., 2021a; Garcia-Rubio et al., 2021b). Tolerant cells are more able to overcome drug pressures relative to the non-tolerant population through enhanced signalling in stress response pathways such as calcium signalling attenuated by the serine/threonine phosphatase calmodulin, HOG, Hsp90 and Tor (Cowen and Steinbach, 2008; Rosenberg et al., 2018). Pharmacological or genetic inhibition of these pathways reduces tolerance to near baseline levels irrespective of initial tolerance level, suggesting a potential role for adjunctive therapies (Cowen et al., 2006; Cowen et al., 2009; Gong et al., 2017). In addition to enhanced signalling, environmental conditions can also select for more highly tolerant strains through increasing cell wall chitin content or enhanced signalling through pathways such as HOG (Lee et al., 2012; Walker et al., 2013).

Given the slow growth of these cells, tolerance is missed by conventional susceptibility assays which generally read the MIC at 24 hours, however measuring at later timepoints (48-72 hours) can demonstrate the degree of strain tolerance. Microbroth dilutions to determine the supra-MIC growth (SMG) or disc assays to measure the fraction of growth (FoG) within the zone of inhibition are two methods for measuring the tolerance of a strain (reviewed in (Berman and Krysan, 2020)). Tolerance has been reported in multiple *Candida* species, mostly in the context of fluconazole (Rosenberg et al., 2018; Kim et al., 2019; Delarze et al., 2020), although tolerance to echinocandins has also been observed in *C. albicans* (Yang et al., 2017), *C. glabrata* (Nagayoshi et al., 2014) and *C. auris* (Nagayoshi et al., 2014). The contribution of tolerance to antifungal treatment failure has yet to be determined: a single study demonstrated an association between highly tolerant *C. albicans* and persistent candidemia following azole treatment (Rosenberg et al., 2018).

There is some overlap between mechanisms of resistance and tolerance. Of note, highly tolerant strains also have lower intracellular azole accumulation (Rosenberg et al., 2018). Like resistance, aneuploidy and loss of heterozygosity have also been identified as mechanisms of tolerance (Selmecki et al., 2009; Healey et al., 2018). The degree of tolerance varies between isolates and is likely due to the intrinsic allele diversity at multiple genetic loci affecting multiple pathways to a differing degree. Tolerance also varies between cells within an isolate, and these are likely due to physiological or metabolic shifts (e.g. cell wall content or iron) that are epigenetically mediated. In the mould *Mucor circinelloides*, RNA interference-dependent epimutations that silence the gene targets of FK506 and rapamycin have been described as mechanisms of tolerance (Calo et al., 2014; Chang et al., 2019). To date, no work has conclusively identified epimutant mechanisms of resistance in *Candida* species, yet it would not be surprising if isolates with unidentified mechanisms of resistance or tolerance contain epimutations parallel to those identified in other species. It remains to be established whether tolerance exists as an adaptive mechanism to slow growth in the presence of drug to provide time for the acquisition of resistance mutation(s) (Cowen and Lindquist, 2005).

Tolerance and heteroresistance highlight the intrinsic ability of fungi to adapt to dual stressors of host immune responses and antifungal drugs beyond our classical understanding of resistance. Further understanding of heteroresistance and tolerance mechanisms, and their relevance in clinical settings, is an important research priority within the field of *Candida* resistance.

## 3 Novel Therapeutic Options for *Candida*

**Figure 3:**
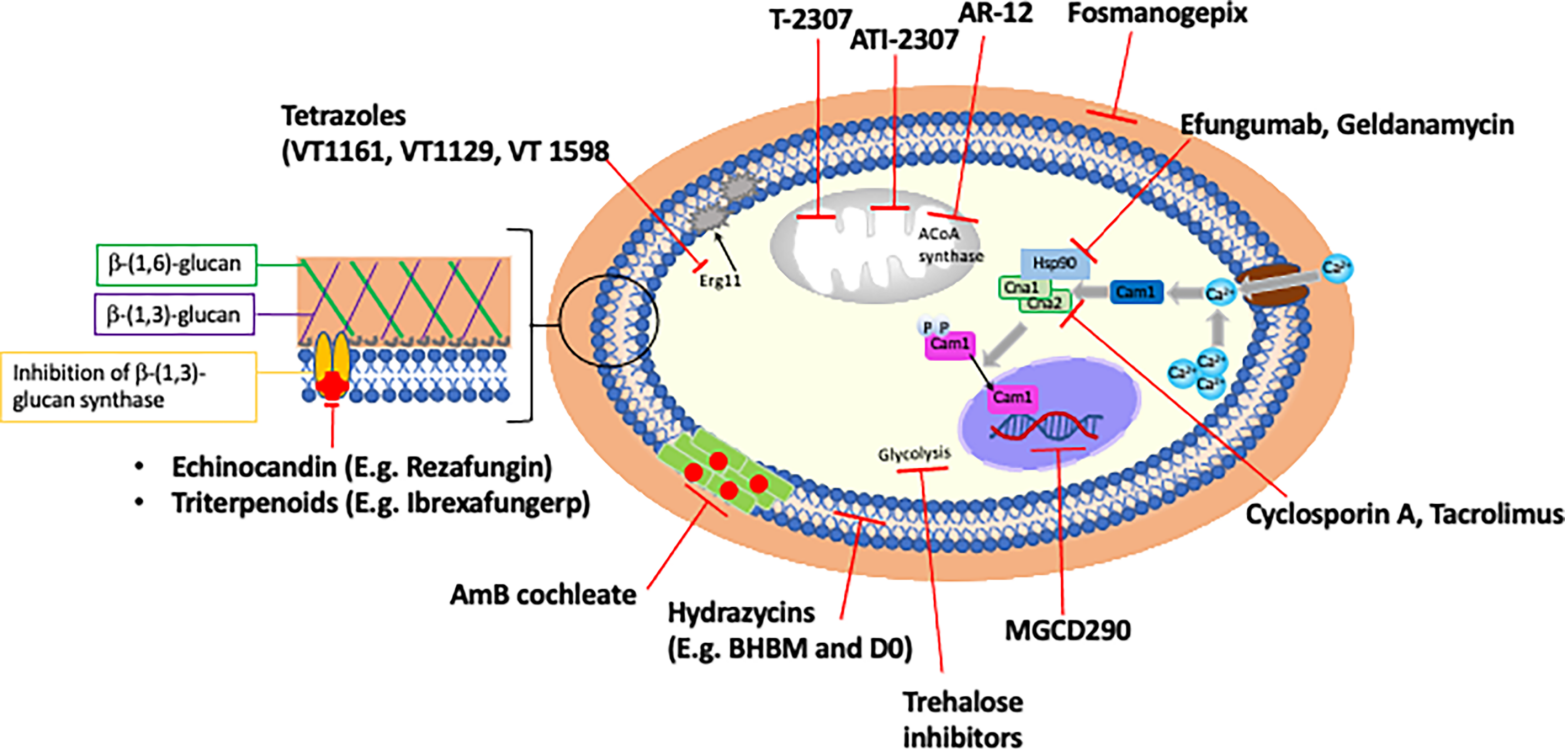
Novel antifungals and potential adjunctive therapies.

Fungi are eukaryotes and share many evolutionarily conserved metabolic pathways with humans, which somewhat restricts available drug targets to pathways essential to fungi only. The recent emergence and increased prevalence of multidrug resistant fungal species has propelled research into novel treatments. Below we give an overview of some of the novel and repurposed compounds with antifungal activity that are at various stages of clinical development, as well as discussing the potential of drug combinations (**Figure 3** and **Table 2**). The list of compounds in this section is comprehensive, but not exhaustive. Compounds were selected for discussion based on either their superior activity against a broad spectrum of resistant *Candida*, for targeting a novel pathway, or being near to clinical deployment with a focus on orally delivered drugs.

**Table 2:** Novel antifungals: target, mechanism of action, spectrum, advantages and stage of development.

Antifungal drug class	Antifungal drug name	Cell target Mechanism of action	Spectrum in resistant Candida spp	Clinical advantages	Stage of development
Tetrazole	VT1129 VT1161 VT1598	Cell membrane Inhibition of Erg11/Cyp51 Inhibition of ergosterol biosynthesis	*C. albicans* *C. glabrata* *C. auris* *C. krusei*	More specific fungal Cyp51 inhibitor; fewer drug interactions; oral	Pre-clinical for IC (Phase I cryptococcal meningitis) Phase III VVC FDA QIDP Phase I
Echinocandin	Rezafungin	Cell wall Inhibition of β-1,3-glucan synthase	*C. albicans* *C. glabrata* *C. krusei* *C. auris*	Superior PK/PD – intermittent dosing penetration (including gut)	Phase III IC and prophylaxis BMT FDA QIDP and fast track aproval
Polyene	MAT2203 (encochleated Amphotericin B)	Organelle membranes Sequesters ergosterol out of cell membrane	*C. albicans* *C. glabrata* *C. krusei* *C. lusitaniae*	Oral; less toxic than IV fromulation	Phase II VVC and CMC FDA QIDP
Triterpenoids	Ibrexafungerp	Cell wall Inhibition of β-1,3-glucan synthase	*C. albicans* *C. glabrata* *C. auris*	Oral; well tolerated; penetrates gut abscesses; separate binding site	Phase III IC, VVC and CMC; *C auris*
N-phosphonooxymethylene	Fosmanogepix	Cell wall Inhibits GPI anchored biosynthesis	*C. albicans* *C. glabrata* *C. auris* *C. parapsilosis*	Novel mechanism of action; Oral; Extensive tissue distribution; Limited cross-resistance	Phase II open label IC/*C auris* FDA fast track approval
Arylamidines	ATI-2307 (formerly T2307)	Mitochondria Collapses mitochondrial membrane potential	*C. albicans* *C. glabrata* *C. krusei* *C. auris*	Novel mechanism of action; Active against biofilms	Phase I
Hydrazycins	BHBM D0, D13	Cell cycle Inhibit vesicular trafficking of sphingolipid precursors	*C. glabrata* *C. krusei*	Novel target	Pre-clinical
Trehalose inhibitors	Tps1 and Tps2 inhibitors	Fungal virulence Inhibition of glycolysis			Compound discovery
Acetyl CoA synthetase inhibitors	AR-12	Disruption of carbon metabolism, histone acetylation, ribosome function, autophagy	*C. albicans* *C. glabrata* *C. krusei*	Novel mechanism of action; Active against resistant species; Well tolerated	Phase I (cancer)
Calcium/calcineurin inhibitors	Cyclosporin A and Tacrolimus (FK506)	Stress response inhibitor Inhibition of calcineurin	*C. albicans* *C. glabrata* *C. krusei*	Inhibit stress-response pathways; fungicidal in combination with current antifungals; abrogate tolerance; non immunosuppressive derivatives developed	Pre-clinical
Hsp90 inhibitors	Efungumab and geldanamycin	Stress response inhibitor Hsp90 inhibitor	*C. albicans*	Inhibit stress response pathways; fungicidal in combination with current antifungals; abrogate tolerance	Phase III IC (enfungumab): not granted EMA approval
Histone deacetylase inhibitors	MGCD290	Nucleus Inhibition of Hos2 and Hsp90	*C. albicans* *C. glabrata* *C. krusei*	Combination with current antifungals; Abrogate tolerance	Phase II VVC
Antibiotics	Colistin	Cell membrane Enhanced ergosterol depletion	*C. albicans*	Combination with current antifungals; abrogate tolerance	Pre-clinical

VVC, vulvovaginal candidiasis; IC, invasive candidiasis; BMT, bone marrow transplant; CMC, chronic mucocutaneous candidiasis; FDA, Federal Drug Administration (USA); QIDP, qualified infectious diseases product; EMA, European Medicine Agency.

### 3.1 New Members of Existing Classes

Although resistance has been identified against the azoles and echinocandins, they are attractive agents as they target proteins unique to fungi. Given that off-target effects and drug-drug interactions are commonly encountered clinical limitations, optimising these drug classes to enhance fungal-specific, on-target activity is an appealing option.

#### 3.1.1 Tetrazoles

Off target effects of azoles *via* their high affinity for haem and non-specific binding to human CYP450 enzymes (Yates et al., 2017) lead to drug-drug interactions with agents metabolised *via* these enzymes as well as effects on liver, skin and vision (Fischer et al., 2005). A new generation of azole-like compounds, the tetrazoles, were rationally designed to target fungal Cyp51 only. A tetrazole metal binding group replaces the triazole and has greater specificity for the fungal lanosterol 14-α demethylase (Erg11) active site over the human Cyp51 isoenzyme (Warrilow et al., 2014; Warrilow et al., 2016).

##### 3.1.1.1 Mechanism

Like the azoles, tetrazoles reversibly and competitively inhibit Erg11p to deplete fungal membranes of ergosterol and disrupt membrane integrity. VT-1129, VT-1598 (quilsecondazole) and VT-1161 (oteseconazole) (Viamet Pharmaceuticals Inc; now Mycovia Pharmaceuticals) were identified as part of a screen for compounds with reduced affinity for human Cyp enzymes (Hoekstra et al., 2014). VT-1161 is more than 1000 times more selective for the *C. albicans* Cyp51 enzyme compared to the human isoenzyme (Warrilow et al., 2014).

##### 3.1.1.2 Activity

VT-1129, VT-1161, and VT-1598 have potent activity against a broad range of *Candida* species, including azole and echinocandin-resistant species, at low MICs (Schell et al., 2017a; Break et al., 2018a).

VT-1161 has a low MIC (0.002 μg/ml) against wild-type, fluconazole-sensitive *C. albicans* (Warrilow et al., 2014) and appears to retain some activity (MIC VT-1161 2 μg/ml) against resistant fluconazole-resistant isolates (MIC FLC 64 μg/ml) and echinocandin-resistant *C. albicans*, *C. glabrata*, *C. krusei* (Garvey et al., 2015; Schell et al., 2017a). In a collection of 68 well-characterised azole-resistant *C. albicans* isolates, susceptibility to VT-1161 was affected by *CDR1* and *MDR1* overexpression and the *erg11* substitutions Y132F, Y132F and K143R, and Y132 and F145L. Other isolates in this collection with point mutations at additional known azole resistance sites retained VT-1161 sensitivity (e.g. Y132H, F145L) (Nishimoto et al., 2019). Overexpression of *CDR1* and *MDR1* reduced susceptibility to VT-1161 in *C. albicans* and *C. glabrata* in another study (Monk et al., 2019).

VT-1161 treatment significantly reduced fungal burden in murine models of vulvovaginal candidiasis (VVC) and IC due to fluconazole-susceptible and -resistant *Candida*. VT-1161 was rapidly absorbed in mouse models and extensively distributed to tissues with rapid penetration to vaginal tissues with a long half-life (>48 hours) (Garvey et al., 2015; Break et al., 2018b).

VT-1598 has the broadest spectrum of activity against fungal species, including fluconazole-resistant *C. albicans* (Break et al., 2018a) and *C. auris* (MIC 0.03-8 μg/ml) (Wiederhold et al., 2019), with enhanced efficacy in neutropenic murine models compared to either fluconazole or caspofungin.

VT-1129 was designed to treat *Cryptococcus*, but also has good *in vitro* activity against many *Candida* species, including azole- and echinocandin-resistant *C. glabrata* and *C. krusei* (Desai et al., 2016; Schell et al., 2017a). No further data on VT-1129 in *Candida* species are available, however the drug has received FDA fast track orphan drug status for treatment of cryptococcal meningitis.

##### 3.1.1.3 Stage of Development

A phase II dose and duration-ranging placebo-controlled randomised trial (NCT02267382) evaluated the efficacy and safety of lower (150mg) and higher (300mg) 12 or 24-week dosing regimens of oral VT-1161 for recurrent VVC (rVVC) (NCT02267382, 2014). The proportion of subjects with ≥1 acute VVC episodes was superior at 0-7% across the 4 VT-1161 arms *vs* 52% in the placebo arm. VT-1161 was well-tolerated with a favourable safety profile and importantly, no evidence of hepatoxicity (Brand et al., 2018). Phase III trials in rVVC (NCT03562156 and NCT03561701, VIOLET, and NCT03840616, ultraVIOLET) are ongoing. A press release on July 29^th^ 2021 reported preliminary findings from the two VIOLET trials in 650 women with efficacy of 90% *vs* 40% (placebo) against recurrence over 48 weeks and excellent tolerability. VT-1161 has received FDA qualified infectious disease product (QIDP) and fast track designation for treatment of rVVC with full approval expected in the US in early 2022.

VT-1598 is undergoing pre-clinical and phase I evaluation for the treatment of *C auris*, cryptococcal meningitis and coccidioidomycosis, whilst development of VT-1129 appears to have been halted.

##### 3.1.1.4 Advantages

The major advantage of the tetrazoles is their enhanced specificity for fungal Cyp51 making this group of drugs more tolerable.

#### 3.1.2 Rezafungin (CD101)

Currently licensed echinocandins (caspofungin, micafungin and anidulafungin) are fungicidal agents against *Candida* species, acting *via* inhibition of the enzyme β-1,3-glucan synthase, causing destabilisation of the cell wall resulting in osmotic instability and cell death. Due to their half-life, echinocandins are given intravenously once daily (24 hours anidulafungin; 9-11 hours caspofungin; 10-17 hours micafungin) (Kofla and Ruhnke, 2011). In addition, the echinocandins have limited penetration into the gastrointestinal tract, the site of *Candida* colonisation as well as intra-abdominal infection, which may have implications for the development of spontaneous resistance.

##### 3.1.2.1 Mechanism

Rezafungin (Cidara Therapeutics) is a novel β-1,3-glucan synthase inhibitor that is a structural analogue of anidulafungin, but with a much longer half-life, facilitating less frequent dosing.

##### 3.1.2.2 Activity

Rezafungin has similar activity compared to other echinocandins with potent *in vivo* pharmacodynamic activity against clinically relevant *Candida* species and greater activity against multidrug resistant strains of *C. auris* than either caspofungin or micafungin (Pfaller et al., 2016; Pfaller et al., 2017b; Arendrup et al., 2018; Lepak et al., 2018b; Lepak et al., 2018a). Rezafungin retains activity at high doses against echinocandin resistant (*fks* mutants) *Candida* species (Berkow and Lockhart, 2018). Rezafungin, but not micafungin, accumulated within intra-abdominal *Candida* abscesses in a mouse model at a level above the mutant prevention concentration of the infecting strain, which may have implications for resistance (Zhao et al., 2017).

##### 3.1.2.3 Stage of Development

Phase I dose-escalation trials in healthy adults demonstrated that rezafungin was well tolerated and safe with a long half-life and high plasma exposures (Sandison et al., 2017). In a phase II randomised trial (STRIVE) comparing two rezafungin weekly dosing regimens to caspofungin (with fluconazole stepdown) for IC, rezafungin was well tolerated, showed rapid *Candida* clearance (19.5h) from blood cultures and comparable overall 14-day cure rates (rezafungin 400mg weekly 60.5%, 400mg/200mg weekly 76.1%, caspofungin 70mg/50mg daily 67.2%) (Thompson et al., 2020).

An ongoing phase III trial (RESTORE NCT03667690) in IC is comparing 14-day global cure and 30-day all-cause mortality between weekly rezafungin and daily caspofungin with fluconazole stepdown. Fungal-free survival with rezafungin as prophylaxis against *Candida*, *Aspergillus*, and *Pneumocytis* infection, compared to oral fluconazole or posaconazole, is currently being evaluated in a phase III trial (ResPECT, NCT04368559) in blood or bone marrow transplantation recipients (Cushion et al., 2016; Ong et al., 2016; Cidara Therapeutics, 2020).

The FDA has granted rezafungin QIDP, fast track and orphan drug status for the treatment of candidemia and invasive candidiasis. The long-term effect of the drug’s PK and its relationship to resistance emergence has yet to be investigated.

##### 3.1.2.4 Advantages

The major advantage of rezafungin over other echinocandins are its superior pharmacokinetics. Rezafungin has increased stability and solubility with more extensive tissue distribution, with higher plasma exposure and greater gut penetration than other echinocandins, with minimal urinary excretion (Sandison et al., 2017; Zhao et al., 2017). Rezafungin has a longer half-life than any echinocandin (80 hours following a single dose, and 150 hours after three doses) allowing for reduced dosing frequency to as low as once weekly (Sandison et al., 2017), facilitating its use in prophylaxis. A higher loading dose of rezafungin improves efficacy through enhanced killing when fungal burden is greatest and reduces the occurrence of spontaneous *fks* mutations compared to either caspofungin or anidulafungin (Locke et al., 2016; Sandison et al., 2017; Lakota et al., 2018). However, cross-resistance has been noted between rezafungin and the other echinocandins (Arendrup et al., 2019).

#### 3.1.3 Encochleated Amphotericin B (MAT2203)

Amphotericin B deoxycholate was the first antifungal licensed in 1959.The drug remains widely used due to its broad spectrum, fungicidal activity with minimal resistance, however its use is compromised by a lack of specificity to fungal sterols resulting in substantial renal toxicity and anaemia (UTZ, 1964; Maddux and Barriere, 1980). Intravenous lipid formulations of amphotericin B have partly but not entirely mitigated these toxicities (Hamill, 2013), and both drugs require intravenous administration. MAT2203 (Matinas BioPharma) is a novel delivery system for AmB deoxycholate consisting of a spiral cochleate lipid bilayer which is orally bioavailable due to its stability in acidic pH (Cuddihy et al., 2019).

##### 3.1.3.1 Mechanism

Like other formulations of amphotericin B, MAT2203 sequesters sterols from fungal membranes. The cochleate is absorbed from the GI tract. Once calcium levels within the cochleate drop sufficiently, the spiral unwinds and releases the drug directly onto fungal cells on contact thereby increasing drug delivery directly onto fungal cells and reducing mammalian cell toxicities (Santangelo et al., 2000). The precise interaction between the cochleate and the fungal cell is not yet fully understood.

##### 3.1.3.2 Activity

The MIC of MAT2203 in *C. albicans* is equivalent to the deoxycholate formulation (0.5 μg/ml) (Zarif et al., 2000). MAT2203 is extensively distributed with good tissue penetration in murine models, where the liver was shown to act as a reservoir for slow release of the drug (Segarra et al., 2002). Mice with systemic candidiasis treated with the encochleated formulation (0.5-20 mg/kg/day) had improved day 16 survival (100%) and reduction in kidney and lung fungal burden relative to intraperitoneal AmB deoxycholeate (70% survival) or liposomal AmB (90% survival) (Santangelo et al., 2000; Hamill, 2013; Shende et al., 2019). No accumulation was observed in healthy mice highlighting the improved fungal specificity of the cochleate formulation.

##### 3.1.3.3 Stage of Development

MAT2203 was well tolerated in healthy volunteers in a single ascending dose phase I study, with mild gastrointestinal adverse events noted (Aigner and Lass-Flörl, 2020). A randomised phase II study (NCT02971007) in women with moderate to severe VVC refractory or intolerant to current therapy yielded disappointing results with MAT2203 (200 mg or 400 mg daily for 5 days) performing poorly compared to a single dose of fluconazole (150 mg): clinical cure at day 12 was significantly lower at 52% and 54.5% compared to 75% for fluconazole. Preliminary data from a small phase II trial (n=16 women) with refractory mucocutaneous candidiasis using higher (400-800mg/d) MAT2203 doses for longer durations (>6 months) suggests all patients eventually achieved a greater than 50% improvement in clinical signs and symptoms and the drug was well tolerated for prolonged periods.

MAT2203 was granted FDA QIDP and fast track status for the treatment of candidiasis in 2015 (BioPharma, 2014).

##### 3.1.3.4 Advantages

Oral administration of the cochleate and its improved tolerability profile hold promise, though comparable efficacy to standard of care oral treatments for mucosal candidiasis has yet to be demonstrated. Molecular umbrella technology is a promising method for the development of further formulations of amphotericin B.

### 3.2 Same Target, New Class

#### 3.2.1 Ibrexafungerp (SCY-078)

##### 3.2.1.1 Mechanism

Ibrexafungerp (Scynexis) also targets β-1,3-glucan synthase, but is structurally distinct from the echinocandins and represents the inaugural member of a novel class of antifungals, the triterpenoids (Gintjee et al., 2020), which bind at an independent site on the enzyme (Schell et al., 2017b; Wring et al., 2017).

##### 3.2.1.2 Activity

Ibrexafungerp has broad spectrum, fungicidal activity against *Candida* species with MIC <2 μg/ml for *C. albicans, C. glabrata, C tropicalis*, and *C. parapsilosis*, but no activity against *C. krusei* and *C. lusitaniae* (Pfaller et al., 2013). Whilst ibrexafungerp retains activity against some echinocandin-resistant *Candida* strains (*fks1/2* mutants), including *C. glabrata* (Jiménez-Ortigosa et al., 2017; Nunnally et al., 2019) and *C. auris* (Berkow et al., 2017; Larkin et al., 2017; Pfaller et al., 2017a; Schell et al., 2017b), presumably due to differential binding on the enzyme, deletion of F625 in *fks1* or F659 in *fks2* were associated with a 40- and 121-fold increase in MIC for ibrexafungerp in *C. glabrata*, respectively. Furthermore, W715L or A1390D substitutions, positioned outside of the hotspot region in *fks2*, increase the MIC to ibrexafungerp by 29 and 20-fold, respectively (Jiménez-Ortigosa et al., 2017).

In murine studies, oral and IV formulations of ibrexafungerp showed extensive distribution and tissue penetration, though not into the CNS (Wring et al., 2017). Ibrexafungerp accumulates at sites with a low pH, with extensive accumulation in vaginal tissue and a necrotic liver abscess in murine models (Larkin et al., 2019; Lee et al., 2020). Neutropenic murine systemic candidiasis models have demonstrated efficacy of ibrexafungerp against *C. albicans, C. glabrata* and *C. parapsilosis*, including against an echinocandin-resistant strain of *C. glabrata* (Lepak et al., 2015; Wring et al., 2017; Wiederhold et al., 2018).

##### 3.2.1.3 Stage of Development

Oral ibrexafungerp was well tolerated in phase I studies even at high doses (up to 1600 mg), with an extensive volume of distribution and good tissue penetration and improved biofilm penetration compared to azoles (Wring et al., 2018; Spec et al., 2019; Azie et al., 2020). A phase II randomised trial (NCT02679456) in rVVC demonstrated superiority of ibrexafungerp relative to fluconazole, with 4-month cure rates of 88% and 65%, respectively, and lower recurrence rates of 4% *vs* 15% (Helou and Angulo, 2017). A phase II dose-ranging acute VVC study (NCT03253094) investigated range of ibrexafungerp dosing regimens compared to single 150 mg dose fluconazole (NCT03253094, 2017), leading onto two recently completed phase III trials of ibrexafungerp 300 mg twice daily for 1 day for the treatment of acute VVC. Ibrexafungerp demonstrated clinical cure rates of 50-63% by day 8-14 against 29-44% with placebo, however gastrointestinal adverse events did occur more commonly in the ibrexafungerp group (VANISH 303 NCT03734991; VANISH 306 NCT03987620). The FDA approved the drug for treatment of VVC in June 2021, marketed as Brexafemme.

A PK study of ibrexafungerp 500 mg or 750 mg as stepdown therapy following IV echinocandin showed that the higher dose would achieve the target PK exposure, was well tolerated and achieved comparable responses to fluconazole (Spec et al., 2019). Two ongoing phase III trials are evaluating ibrexafungerp fungal diseases (including mucocutaneous and invasive candidiasis) refractory to or intolerant of standard antifungal treatment (FURI, NCT03059992, target n=200) and in treating invasive *C. auris* infection (CARES, NCT03363841). Ibrexafungerp has been given QIDP status by the FDA for invasive candidiasis.

##### 3.2.1.4 Advantages

Advantages of ibrexafungerp over the echinocandins include oral administration, penetration into intraabdominal abscesses and retained activity against some echinocandin resistant isolates.

### 3.3 Novel Mechanism of Action

In the context of emerging resistance, the development of antifungals with a novel mechanism of action against a fungal-specific pathway and/or potentiating the activity of the current antifungals is a priority for the research community and the pharmaceutical industry. Finding novel targets that are unique to fungi has been a challenge given that up to 80% of hits turn out to be false positives (Pouliot and Jeanmart, 2016) alongside limited incentives for pharmaceutical investment in antifungal drug development due to the comparatively lower number of patients with invasive fungal infection compared to bacterial infection. In recent years, several novel agents in the pipeline targeting unique pathways give grounds for hope.

#### 3.3.1 Fosmanogepix

##### 3.3.1.1 Mechanism

Fosmanogepix (APX001 and E1211, Amplyx Pharmaceuticals) is the inaugural member of the N-phosphonooxymethylene prodrugs that is efficiently converted to manogepix (APX001A and E1210), an inhibitor of fungal glycosylphosphatidylinositol (GPI) proteins. Manogepix was identified in a screen for compounds that interfere with the correct localisation of cell wall GPI-anchored mannoproteins (Tsukahara et al., 2003). Manogepix is highly specific for fungal, but not human Gwt1, an enzyme that catalyses the acetylation of inositol, an essential step in the early stages of GPI anchor biosynthesis (Miyazaki et al., 2011; Watanabe et al., 2012). Gwt1 is essential for trafficking and anchoring mannoproteins to the cell wall and outer cell membrane to maintain cell wall integrity, adhesion, pathogenicity, and evasion of the host immune system. Inhibition of Gwt1 prevents cell wall reinforcement thereby reducing hyphal formation, virulence, germ tube formation, and biofilm formation, in addition to inducing morphological changes to the cell size and shape resulting in exposure of β-1,3-glucan to host immune cells and ER stress (Watanabe et al., 2012).

##### 3.3.1.2 Activity

Fosmanogepix is highly active against many *Candida* species: MIC_90_ *C. albicans* (0.008-0.06 μg/ml), *C. glabrata* (0.06-0.12 μg/ml), *C. auris* (0.03 μg/ml) (Hata et al., 2011; Miyazaki et al., 2011; Pfaller et al., 2011a; Watanabe et al., 2012; Hager et al., 2018; Zhao M. et al., 2018), though lacks activity against *C. krusei* (MIC_90_ ≥ 0.5 μg.ml) (Miyazaki et al., 2011). No cross-resistance has been reported between the echinocandins, amphotericin B and fosmanogepix for multiple *Candida* species (Miyazaki et al., 2011; Pfaller et al., 2011a; Wiederhold et al., 2015b; Zhao, Y et al., 2018), with most studies reporting no cross-resistance to fluconazole-resistant *erg11* mutants (Miyazaki et al., 2011; Pfaller et al., 2011a). There does however appear to be a correlation with fluconazole MICs, with a 2-8-fold increase in MIC to fosmanogepix reported in a subset of fluconazole-resistant *Candida* isolates (Arendrup et al., 2018; Arendrup and Jørgensen, 2020). A recent study of the mechanism of reduced susceptibility to both fosmanogepix and fluconazole identified increased efflux through Cdr11 and Snq2 as a result of a gain-of-function mutation in the transcription factor *zcf29* in *C. albicans* and a mitochondrial DNA deletion activating *MDR1* expression in *C. parapsilosis* (Liston et al., 2020). Despite MIC increases, the MIC of these isolates remained low. Further studies are needed to determine the clinical relevance of these findings and to explore the link with azole cross-resistance *via* efflux.

*In vitro* serial passage showed an 8-fold rise in MIC in *C. albicans* (18 passages) and *C. parapilosis* (3 passages), with no increase in MIC for *C. glabrata*, *C. auris* and *C. tropicalis* (Kapoor et al., 2020). GWT sequencing of isolates with reduced susceptibility demonstrated a single valine to alanine point mutation in *gwt* (V163A *C. glabrata*; V162A *C. albicans*) which appears to be essential for manogepix binding in all Gwt orthologs. Neither mutation affected susceptibility to echinocandins or azoles. A further study identified enhanced efflux as the mechanism of fosmanogepix resistance in *C. parapsilosis* and *C. albicans* that have a 4-8 fold increase in MIC that were not associated with mutations in the *GWT* gene (Liston et al., 2020).

Fosmanogepix is effective at reducing fungal burden and improving survival in immunosuppressed murine models using wild type, azole- and echinocandin-resistant *Candida*, including *C. auris* (Hata et al., 2011; Miyazaki et al., 2011; Wiederhold et al., 2015b; Hager et al., 2018; Zhao et al., 2018). Rodent and primate studies have demonstrated good oral bioavailability and safety and extensive tissue penetration (liver, lungs, spleen, brain, kidney, eye) (Hata et al., 2011; Mansbach et al., 2017). In addition, fosmanogepix has demonstrated synergy with the azoles and echinocandins in animal models (Hata et al., 2011).

##### 3.3.1.3 Stage of Clinical Development

Fosmanogepix has undergone four phase I trials (NCT03333005; NCT04166669; NCT02956499; NCT02957929) using intravenous and oral administration, showing a low propensity for drug-drug interactions, good tolerability and no serious adverse effects reported in healthy volunteers (Hodges et al., 2017a; Hodges et al., 2017b), with findings in acute myeloid leukaemia patients not yet reported.

In an open label phase II trial (NCT03604705), with a minimum of 3 days’ IV therapy followed by oral stepdown, in non-neutropenic patients (n=20) with suspected or confirmed candidemia, 16/20 patients achieved 14-day treatment success (composite of alive, two negative blood cultures and no rescue antifungal treatment). In a phase II trial in *C. auris* candidemia or IC (NCT04148287, APEX), 9 participants received IV fosmanogepix on days 1-3 followed by oral fosmanogepix for up to 42 days. However, this trial was terminated early due to COVID19. Fosmanogepix has been granted FDA fast track approval for 7 separate indications including invasive candidiasis.

##### 3.3.1.4 Advantages

Fosmanogepix exploits a novel mechanism of action that has limited potential for cross-resistance. It is broad spectrum, effective in animal and early-stage clinical trials, is orally bioavailable and well tolerated, and shows promise for use in infections due to multidrug resistant species such as *C. auris*.

#### 3.3.2 ATI-2307

##### 3.3.2.1 Mechanism

ATI-2307 (Appili Therapeutics), formerly T-2307 (developed by Toyama Chemical Co), is an arylamidine similar to pentamidine. Its precise mechanism of action remains elusive, however it is selectively taken up into fungal cells *via* the spermidine transport system (Nishikawa et al., 2010) and initiates the collapse of mitochondrial membrane potential, ultimately inhibiting respiration and energy production resulting in fungicidal activity in *C. albicans* and *C. krusei*, but fungistatic activity in *C. glabrata* and *C. parapsilosis* (Nishikawa et al., 2010; Shibata et al., 2012; Yamashita et al., 2019).

##### 3.3.2.2 Activity

ATI-2307 is active against a broad spectrum of *Candida* species including azole and echinocandin-resistant species and *C. auris* (Mitsuyama et al., 2008; Yamada et al., 2010; Wiederhold et al., 2015a; Wiederhold et al., 2016; Wiederhold et al., 2020) with lower *in vitro* MICs (0.0005-0.125 μg/ml) compared to azoles, micafungin, and amphotericin B (Mitsuyama et al., 2008). ATI-2307 exerted a more potent effect at equivalent doses of amphotericin B or micafungin in immunocompetent murine models of systemic wild type *C. albicans* and echinocandin resistant infections (Mitsuyama et al., 2008). ATI-2307 also reduced kidney fungal burden and improved survival in mice infected with *C. auris* (Wiederhold et al., 2020), as well as neutropenic mice infected with *C. glabrata* harbouring the Fks2 substitution R1379S (Wiederhold et al., 2016). ATI-2307 had minimal effect on mitochondrial morphology and potential of rat liver cells (Nishikawa et al., 2010; Yamada et al., 2010; Nishikawa et al., 2016).

##### 3.3.2.3 Stage of Development

One report states that ATI-2307 has completed a phase I study in the USA with no adverse effects noted (Nishikawa et al., 2017), however these data are not currently publicly available.

##### 3.3.2.4 Advantages

Although this compound remains in the early stages of clinical development, ATI-2307 shows promise as a fungal-selective compound (Nishikawa et al., 2010; Nishikawa et al., 2016) and *in vitro* and *in vivo* data highlight its potential in treating a range of *Candida* species including more resistant isolates.

#### 3.3.3 Hydrazycins (BHBM, D0, D13)

Sphingolipid biosynthesis is essential for eukaryotic metabolism as well as fungal pathogenicity (Noble et al., 2010; Oura and Kajiwara, 2010) thus represents an attractive new antifungal target given the structural disparity between fungal and mammalian sphingolipids (Mor et al., 2015; Rollin-Pinheiro et al., 2016). Glucosylceramide is critical for fungal progression through the cell cycle and growth at neutral and alkaline pH (Saito et al., 2006), characteristic of human blood and cerebrospinal fluid. Antibodies that inhibit glucosylceramide have demonstrated extensive antifungal effects both *in vitro* and *in vivo* but lack specificity for the fungal sphingolipid (Rodrigues et al., 2007).

##### 3.3.3.1 Mechanism

The hydrazycins, (E)-N′-(3-bromo-6-hydroxybenzylidene)-2-methylbenzohydrazide (BHBM) and benzohydrazide (D0) were identified in a screen for compounds that specifically inhibit the biosynthesis of fungal glucosylceramide. The hydrazycins inhibit the vesicular trafficking of ceramide, a precursor lipid of glucosylceramide, thereby halting glucosylceramide and sphingolipid biosynthesis and disrupting cell division (Mor et al., 2015).

##### 3.3.3.2 Activity

BHBM has variable activity against *Candida* species with moderate activity against *C. krusei* and *C. glabrata*, (MIC 2-32 μg/ml), but poor activity against *C. albicans and C parapsilosis* (MIC>32 μg/ml) (Mor et al., 2015). A derivative of BHBM, D13, was selected from a screen for enhanced specificity to the fungal target activity and activity, with an MIC of 1 μg/ml in *C. albicans* and *C. krusei* (Lazzarini et al., 2018). Synergy of D13 with fluconazole was reported for one of two *C. krusei* strains (FICI 0.31), and with caspofungin for both isolates, but neither combination was synergistic for fluconazole-sensitive or -resistant *C. albicans* (Lazzarini et al., 2018).

Two murine systemic candidiasis model studies evaluated the *in vivo* activity of the hydrazycins. One reported a 75% and 62.5% 21-day survival in the D0 and BHBM arms, respectively (Mor et al., 2015); the other a much lower 20% day 21 survival with either D13 or BHBM against 0% with fluconazole (Lazzarini et al., 2018).

##### 3.3.3.3 Stage of Development

The hydrazycins remain in pre-clinical development. Sphingolipid metabolism is being explored separately for use in fungal vaccines.

##### 3.3.3.4 Advantages

BHBM, D0, and D13 are fungal-specific with a novel sphingolipid target. Although current hydrazycins do not show sufficient spectrum against clinically relevant *Candida* species, the BHBM derivative, D13, represents an improvement and all congeners have all been reported to re-sensitise some azole-resistant species to azoles. Screens for additional daughter compounds of BHBM may identify novel hydrazycins with superior antifungal activity.

#### 3.3.4 Trehalose Inhibitors

Trehalose is a two glucose non-reducing sugar cleaved to generate glucose for glycolysis. The trehalose pathway is of interest due to its fungal specificity and role in fungal growth and virulence, with trehalose critical for fungal survival at high temperatures, acting as an antioxidant under oxidative stress and protecting against other host-induced stressors through interactions with proteins and phospholipids that reinforce the cell wall and prevent degradation of cell membrane and intracellular proteins (Iturriaga et al., 2009).

Although relatively few trehalose-inhibiting compounds have been identified to date, genetic deletion of either of the primary synthesising enzymes in this pathway (Tps1 (trehalose-6-phosphate synthase) and Tps2 (trehalose-6-phosphate phosphatase)) have been identified as essential for *C. albicans* infectivity in mammalian studies, preventing hyphal development and macrophage survival (Zaragoza et al., 1998; Van Dijck et al., 2002; Zaragoza et al., 2002; Martínez-Esparza et al., 2007). Tps2 inhibition results in the accumulation of its substrate trehalose-6-phosphate, which is likely toxic to the fungus at very high concentrations (Perfect et al., 2017). Uniquely for phosphatases, inhibition of Tps2 was not associated with any off target activity to other phosphatases (Perfect et al., 2017). Drug discovery of Tps1 and Tps2 inhibitors is underway *via* two major strategies. Purified Tps1 and Tps2 enzymes are being used to identify inhibitors in a high throughput Transcreener UDP fluorescence polarisation assay (Perfect et al., 2017). Several compounds were identified with activity against *Cryptococcus*, however murine studies were unfavourable: screens for novel trehalose inhibitors against *Candida* species are underway. The crystal structures for both Tps1 and Tps2 were recently solved (Miaoa et al., 2016) and are now guiding structure-activity compound design.

Further characterisation in *Candida* infections and the development of trehalose pathway inhibitors pose an interesting new class of antifungal compounds with potential as treatments of tolerance.

#### 3.3.5 Turbinmicin

Turbinmicin is a promising new antifungal that was recently discovered in a high-throughput screen of bacteria isolated from the microbiome of the sea squirt. Turbinmicin belongs to the group of highly oxidised type II polyketides and is produced by *Micromonospora* species. Genetic knockdown and haploinsufficiency screens in *S. cerevisiae*, along with the disruption of ER-Golgi vesicular transport to the plasma membrane identified the essential vesicle transport protein Sec14 as the most likely target (Zhang et al., 2020). Many *Candida* rely on the vesicular delivery of extracellular matrix components to form extensive biofilms for drug resistance. Turbinmicin is, therefore, particularly promising as an anti-biofilm drug through interference with the assembly of the extracellular matrix: a rat central venous catheter *C. albicans* biofilm model demonstrated an almost complete elimination of biofilm in turbinmicin treated rats relative to buffer treated controls (Zhao et al., 2021).

Turbinmicin demonstrated broad spectrum, fungicidal activity *in vitro*, including against pan-resistant *C. auris*, MDR *C. glabrata* and triazole-resistant *Aspergillus fumigatus*, with MICs of 0.5 μg/ml or less (Zhang et al., 2020). Furthermore, a combination of fluconazole and turbinmicin was more efficacious at eliminating biofilm than either drug alone, possibly due to turbinmicin disrupting the extracellular matrix sufficiently to render cells more vulnerable to the azole (Zhao et al., 2021). *In vitro* safety studies in human red blood cells identified that turbinmicin did not trigger haemolysis even at concentrations 1000 times the MIC. Turbinmicin treatment resulted in a 3.6 log reduction of fungi relative to control vehicle in a disseminated candidiasis mouse model of pan-resistant *C. auris*, with good tolerability across a range of doses up to 256 mg/kg/d (Zhang et al., 2020).

Turbinmicin would represent the inaugural antifungal to target Sec14, although this protein has similarity to the *hs*Sec14 so further analysis for potential cross-reactivity would be prudent. Further preclinical development will entail studies in additional mammalian species and safety monitoring over longer treatment courses.

### 3.4 Repurposed

Given the significant resources required for development and clinical validation of novel compounds, a favoured approach is to search for antifungal activity in previously approved non-antifungal therapeutics, which can then potentially be modified to reduce undesirable off-target effects. Large scale, high-throughput screens have identified some promising therapeutic avenues, however *in vitro* efficacy has not always translated into clinical efficacy.

#### 3.4.1 AR-12

AR-12 (Arno Therapeutics) is a celecoxib derivative that was initially developed as an anticancer protein kinase inhibitor, but also has fungicidal activity at low doses against a range of *Candida* isolates, including *C. albicans* biofilms (Baxter et al., 2011; Koselny et al., 2016b).

##### 3.4.1.1 Mechanism

Whilst the anticancer properties of AR-12 are attributed to its kinase inhibition, its antifungal activity is *via* two distinct routes. Firstly, by the specific inhibition of the fungal acetyl CoA (Acs2p) which has a multitude of effects due to the vast range of processes that acetyl CoA is involved with (e.g. carbon metabolism, histone acetylation, ribosome function and autophagy) (Koselny et al., 2016b). Inhibition of Acs2p ultimately induces cell lysis. Secondly, AR-12 also enhances the host antifungal immune response through down-regulation of host chaperone proteins such as Grp89 and Hsp90, although the precise details have not yet been determined (Koselny et al., 2016b).

##### 3.4.1.2 Activity

AR-12 is fungicidal against *Candida* species (MIC *C. albicans, C. glabrata, C. parapsilosis, C. tropicalis, C. krusei* 2-4 μg/ml) and retains activity against strains with intermediate or resistant fluconazole MICs (MIC > 128 μg/ml) due to gain-of-function mutations affecting efflux pump activity (Koselny et al., 2016a). In addition, deletion of neither TAC1 nor MRR1 affected susceptibility of isolates to AR-12. Combination of AR-12 with fluconazole re-sensitised some fluconazole-resistant *C. albicans* and *C. glabrata* strains. AR-12 remained active against echinocandin-resistant strains containing FKS mutations and synergised with caspofungin in caspofungin-resistant strains of *C. glabrata* (Koselny et al., 2016a).

##### 3.4.1.3 Stage of Development

Early phase I studies (NCT00978523) for the anticancer activity of AR-12 identified good serum concentrations with limited adverse effects, however development was halted in 2017 when Arno Therapeutics declared bankruptcy (Koselny et al., 2016b).

##### 3.4.1.4 Advantages

AR-12 has a broad antifungal spectrum in yeasts and moulds and has been shown to be well tolerated in Phase I human clinical trials at doses relevant for antifungal activity. Commonly occurring mechanisms of azole and echinocandin resistance do not appear to affect susceptibility to AR-12. Combinations with existing antifungals may show potential in tackling drug-resistant candidiasis.

### 3.5 Combination/Adjunctive Therapies

Combination therapy using drugs with distinct targets, including drugs without direct antifungal activity, holds appeal in terms of potentially faster fungal clearance and reduction of resistance emergence, prolonging the longevity of the current antifungal arsenal. This needs to be evaluated carefully and balanced against additional cost and potentially additive toxicities.

#### 3.5.1 Flucytosine (5FC)

5FC is an old antifungal with good oral bioavailability, excellent tissue penetration and fungicidal activity against *Candida* species (Pfaller et al., 2012), with a target distinct from the widely used cell wall and membrane-acting agents. Secondary resistance develops if used as monotherapy, hence the agent is always used in combination, with currently a niche role, given with amphotericin B in *Candida* meningitis or endocarditis (Perfect et al., 2010). 5FC has not been widely used to treat candidaemia/IC due to concern regarding its toxicity, namely bone marrow suppression due to its metabolite 5-fluorouracil (5FU). These side effects were more pronounced at the historically higher dosage of 150 (or even 200) mg/kg/day (Francis and Walsh, 1992). More recently however, in the treatment of cryptococcal meningitis, a lower dose of 100 mg/kg/day (25mg/kg four times a day) given for 14 days was well tolerated and enhanced fungal clearance when used in combination with either fluconazole or amphotericin B (Van Der Horst et al., 1997; Day et al., 2013; Molloy et al., 2018).

PK/PD studies have demonstrated that the activity of 5FC (as opposed to toxicity) is concentration-independent, and time-dependent (Hope et al., 2006; Brouwer et al., 2007; Lepak and Andes, 2015). Given the lower MICs for *Candida* species (MIC_90_ ~1 μg/mL for *C. albicans*, as opposed to 8-16 μg/mL for *Cryptococcus*), lower doses of 25-50mg/kg/day may be sufficient to achieve maximal fungicidal activity against most *Candida* species. A recent extensive *in vitro* screen for combinations of licensed antifungals against a large *C. auris* isolate collection identified synergy when 5FC was combined with echinocandins or amphotericin B, with combinations of anidulafungin or micafungin with 1mg/L 5FC effectively inhibiting echinocandin-resistant *C. auris* isolates (O’Brien et al., 2020).

The drug is also limited by its four times daily dosing requirement, however a slow-release formulation has been developed in conjunction with DNDi (Drugs for Neglected Diseases initiative) and is entering phase I trials. Combinations of 5FC with azoles and echinocandins as well as the novel anti-*Candida* agents warrant clinical exploration, particularly in the treatment of IC due to drug resistant *Candida* species.

#### 3.5.2 Calcineurin and Hsp90 Inhibitors

The serine/threonine phosphatase calcineurin is a conserved regulator of calcium homeostasis in eukaryotes and activates many target genes with a variety of cellular functions, including fungal growth, morphological transition, cell wall integrity and host survival (Sanglard et al., 2003; Reedy et al., 2010; Juvvadi et al., 2014; Juvvadi et al., 2017). Calcineurin is essential for fungal adaptation to multiple environments, including survival to antifungal drugs, with resistance in many strains linked to the calcium-calcineurin pathway (Brand et al., 2007; Zhang et al., 2012). Furthermore, inhibitors of calcineurin (e.g. FK506 and cyclosporin) synergistically enhance the antifungal properties of fluconazole, rendering fungicidal activity against *C. albicans* (Marchetti et al., 2000a; Cruz and Goldstein, 2002; Onyewu et al., 2003). Deletion of either of the catalytic or regulatory subunits of calcineurin in *C. albicans* enhances susceptibility to multiple stressors, including disruptors of the cell membrane (e.g. azoles) or cell wall (e.g. echinocandins), serum and alkaline pH, and is associated with hypovirulence in murine models of disseminated candidiasis (Cruz and Goldstein, 2002; Bader et al., 2003; Sanglard et al., 2003; Bader et al., 2006), though not in vaginal candidiasis (Bader et al., 2006). Therefore, inhibition of calcineurin signalling to disrupt fungal virulence represents an attractive adjunctive therapy option.

##### 3.5.2.1 Mechanism

External stressors induce an influx of calcium ions into the fungal cytoplasm, which bind to the calcium-binding protein calmodulin. Calcineurin binds to the calcium-calmodulin complex inducing a conformational change in the phosphatase that removes the autoinhibitory domain, thus activating calcineurin. Calcineurin transmits calcium signals *via* the dephosphorylation and subsequent nuclear translocation of the transcription factor Crz1 which in turn activates the transcription of calcineurin-dependent genes involved in cellular signalling, growth, vesicular trafficking, and cell wall integrity (Karababa et al., 2006).

FK506 and cyclosporin bind to immunophilins forming a potent calcineurin inhibitor complex thereby preventing the activation of its phosphatase activity and subsequent activation of Crz1 (Liu et al., 1991; Thewes, 2014).

##### 3.5.2.2 Activity

Calcineurin inhibitors abrogate tolerance in *Candida* and enhance the antifungal activity of current antifungals: Combination of a calcineurin inhibitor with fluconazole renders fungicidal activity in many *Candida* species with varying levels of tolerance (Marchetti et al., 2000a; Marchetti et al., 2000b; Cruz and Goldstein, 2002; Onyewu et al., 2003). *In vitro* screens against *C. albicans*, *C. glabrata*, and *C. krusei* using drugs for non-mycological conditions with antifungals identified cyclosporin A and FK506 (tacrolimus) as synergistic with both azole and non-azole inhibitors of ergosterol biosynthesis (e.g. terbinafine). However, cyclosporin A and FK506 are potent immunosuppressants typically prescribed following solid organ transplantation and could thus enhance host susceptibility to *Candida* infection. FK506 analogues that lack immunosuppressive activity yet retain synergy with antifungals have demonstrated promising *in vitro* and *in vivo* results (Lee et al., 2018; Jung and Yoon, 2020).

Calcineurin activity can also be inhibited through depletion of the molecular chaperone heat shock protein 90 (Hsp90). Hsp90 is implicated in the emergence of resistance to fluconazole and echinocandins in *C. albicans* and *C. glabrata* and potentiates its activity through binding to the catalytic subunit of calcineurin (Cowen and Lindquist, 2005; Cowen et al., 2006). Hsp90 inhibitors (e.g. geldanamycin and efungumab) induce the degradation of the downstream protein calcineurin and also synergise with azoles and echinocandins *in vitro* (Singh et al., 2009; Singh-Babak et al., 2012). Moreover, Hsp90 represents a favourable target as it interacts with approximately 10% of the proteome, therefore inhibition could disrupt multiple essential pathways (McClellan et al., 2007). Efungumab (previously Mycograb) is a monoclonal antibody targeting Hsp90 developed by NeuTec Pharma and subsequently acquired by Novartis. Mycograb combined with a lipid formulation of AmB was reported as resulting in faster fungal clearance, improved d10 response rates and reduced mortality in a phase III trial in IC compared to AmB alone (Pachl et al., 2006). However, the study methodology was questioned (Herbrecht et al., 2006) and the European Medicines Agency has subsequently twice refused marketing authorisation for Mycograb, citing product safety and quality issues. Hsp90 inhibitors are currently in development as anti-cancer drugs, however these are precluded from human use due to host toxicities, but recent identification of specific inhibitors of the *C. albicans* Hsp90 nucleotide binding domain offer a path towards fungal selectivity (Whitesell et al., 2019).

##### 3.5.2.3 Advantages

Calcineurin and Hsp90 are promising targets due to their central role in many fungal growth and invasion pathways, priming host cells to antifungal drugs and antifungal immunity. Combinations of azoles with inhibitors or these pathways renders azoles fungicidal and may limit the development of resistance. Calcineurin and Hsp90 are both structurally highly conserved throughout fungal species offering an opportunity for development of a broad-spectrum, non-immunosuppressive derivative of currently licensed inhibitors.

#### 3.5.3 MGCD290

Histone deacetylases (HDACs) deacetylate lysine residues on histones and cellular proteins, thereby controlling transcription, cell proliferation and cell motility. Again, this class of drugs are cytotoxic and are in use as anti-cancer agents (Eckschlager et al., 2017).

##### 3.5.3.1 Mechanism

HDAC inhibitors (trichostatin A, apicidin and sodium butyrate) induce apoptosis and cell cycle arrest (Grozinger and Schreiber, 2002). MGCD290 (MethylGene, Mirati Therapeutics) inhibits the fungal histone deacetylase 2 (Hos2) with an additional target through inhibition of the deacetylation of fungal Hsp90, involved in fungal stress adaptation (Robbins et al., 2012) and may have a role in addressing fungal tolerance. Like, MGCD290, Trichostatin A reduced tolerance and inhibited upregulation of *C. albicans ERG11* and *CDR* when co-administered with fluconazole, itraconazole and terbinafine (inhibitors of sterol synthesis), but had little effect on a fluconazole-resistant isolate (Smith and Edlind, 2002).

##### 3.5.3.2 Activity

MGCD290 only has modest antifungal activity against *Candida* species as monotherapy (MIC 0.5–16 μg/ml for *C. albicans*, *C. glabrata*, *C. krusei*), however synergises with azoles and echinocandins at low doses in susceptible as well as azole- and some echinocandin- resistant *Candida* species (Pfaller et al., 2009; Pfaller et al., 2015). MGCD290 in combination with fluconazole increased survival and significantly reduced fungal burden in the kidney in murine and rat models of systemic candidiasis relative to fluconazole alone (Besterman et al., 2015).

##### 3.5.3.3 Stage of Development

Four phase I trials in healthy volunteers of oral MGCD290 alone or in combination with fluconazole for 14 days demonstrated a good safety profile and favourable PK (Besterman, 2012). Despite compelling *in vitro* data, MGCD290’s promising antifungal activity failed to translate: a phase II trial of MGCD20 in combination with fluconazole for VVC showed no significant benefit of the combination over fluconazole alone (NCT01497223).

##### 3.5.3.4 Advantages

MGCD290 is an oral agent with a novel target demonstrating potential *in vitro* through its impact on tolerance, but whose clinical potential has not been realised. Inhibition of Hsp90 through preventing its deacetylation is potentially more attractive given the immunosuppressive consequences of direct Hsp90 pharmacological inhibition.

#### 3.5.4 Colistin

Colistin (polymyxin B) is a positively charged lipopeptide that is bactericidal through binding of membrane lipids and used in treatment of highly resistant gram-negative bacteria but is associated with significant nephrotoxcicity. Although colistin itself has minimal antifungal activity in *Candida* species (Zhai et al., 2010), it is fungicidal when combined with low doses of fluconazole, an effect that is particularly pronounced in highly azole tolerant strains (Bibi et al., 2021). Colistin enhances the ergosterol-depleting activity of fluconazole through binding to membrane lipids (PS, PI, PE), which was particularly pronounced in ergosterol-depleted cells following fluconazole treatment or genetic knockout of *erg11 erg3 erg24*. The combination was superior to fluconazole monotherapy in the *Galleria mellonella* model infected with highly tolerant *C. albicans*. Colistin also synergises with echinocandins in echinocandin-sensitive, but not -resistant *C. albicans* (Zeidler et al., 2013) and with caspofungin, but not micafungin in *C. auris* (Bidaud et al., 2020). It is currently unclear whether colistin has synergy with polyenes or with fluconazole in non-*albicans Candida* species.

## 4 Conclusion

With the increasing use of antifungal agents in prophylaxis, empiric, or targeted treatment of invasive candidiasis in an ever-expanding range of susceptible hosts, *Candida* species have evolved a myriad of resistance mechanisms to established antifungals. For example, poor response to the fungistatic azoles, where either heteroresistance and tolerance may preclude the development of resistance, is most frequently associated with SNPs or aneuploidy-mediated mechanisms that increase the amount of target protein, alter the conformation of the drug binding site, or act to reduce intracellular drug accumulation.

Following slow progress since the early 2000s when the echinocandins were developed, the past decade has witnessed exciting developments and clinical trial progression of novel anti-*Candida* drugs based on different binding sites on the same target (ibrexafungerp) or completely new targets (fosmanogepix), as well as improved formulations of existing compounds (encochleated amphotericin B, rezafungin, tetrazoles). Other advantages of many novel agents are oral formulations, broad-spectrum anti-*Candida* activity including effectiveness against drug-resistant *Candida* species and a lack of cross-resistance with established antifungals. A number of repurposed agents with minimal intrinsic antifungal activity may hold promise as adjunctive therapies providing they can be reconfigured to minimise their off-target effects and toxicities. The old agent flucytosine is an under-utilised partner drug in the treatment of invasive candidiasis, including drug-resistant infections. Additional pathways emerging as promising target pathways have been well reviewed elsewhere (Nguyen et al., 2021).

Future priorities for academia are to better understand the mechanisms of resistance for the novel antifungal drug classes and how these might best be deployed in the clinic to prevent the development of resistance, including optimising PK/PD against resistant species and combination therapy with drugs with distinctive antifungal targets or mechanisms of action.

## Author Contributions

SM and TB conceived the Review. SM wrote the first draft, with input from TB. Both authors contributed to the final draft. All authors contributed to the article and approved the submitted version.

## Funding

St. George’s Hospital Charity, grant ref = 19-20 001, Emerging Leaders Prize in Antimicrobial resistance from the Medical Research foundation, grant ref = MRF-160-0009-ELP-BICA-C0802.

## Conflict of Interest

TB has received Speaking fees from Gilead Sciences and Pfizer and research funding from Gilead Sciences unrelated to the submitted work.

The remaining author declares that the research was conducted in the absence of any commercial or financial relationships that could be construed as a potential conflict of interest.

## Publisher’s Note

All claims expressed in this article are solely those of the authors and do not necessarily represent those of their affiliated organizations, or those of the publisher, the editors and the reviewers. Any product that may be evaluated in this article, or claim that may be made by its manufacturer, is not guaranteed or endorsed by the publisher.
